# Cognitive Processes in Decisions Under Risk are not the Same as in Decisions Under Uncertainty

**DOI:** 10.3389/fnins.2012.00105

**Published:** 2012-07-12

**Authors:** Kirsten G. Volz, Gerd Gigerenzer

**Affiliations:** ^1^Werner Reichardt Centre for Integrative NeuroscienceTuebingen, Germany; ^2^Adaptive Behavior and Cognition, Max Planck Institute for Human DevelopmentBerlin, Germany

**Keywords:** as-if versus process models, neuroscience of decision making, risk and uncertainty, small world versus large world problems, heuristics

## Abstract

We deal with *risk* versus *uncertainty*, a distinction that is of fundamental importance for cognitive neuroscience yet largely neglected. In a world of risk (“small world”), all alternatives, consequences, and probabilities are known. In uncertain (“large”) worlds, some of this information is unknown or unknowable. Most of cognitive neuroscience studies exclusively study the neural correlates for decisions under risk (e.g., lotteries), with the tacit implication that understanding these would lead to an understanding of decision making in general. First, we show that normative strategies for decisions under risk do not generalize to uncertain worlds, where simple heuristics are often the more accurate strategies. Second, we argue that the cognitive processes for making decisions in a world of risk are not the same as those for dealing with uncertainty. Because situations with known risks are the exception rather than the rule in human evolution, it is unlikely that our brains are adapted to them. We therefore suggest a paradigm shift toward studying decision processes in uncertain worlds and provide first examples.

## Risk ≠ Uncertainty

In 1999, Elkhonon Goldberg and Kenneth Podell distinguished between adaptive and veridical decision making. Noticing the predominance of the latter in the cognitive neuroscientific studies at that time, they concluded that new paradigms were desperately needed:

In a typical experimental paradigm used in cognitive neuroscience, one possible response is correct and others are incorrect. The determination of what is correct and what is “incorrect” is inherent in the experimental situation (external milieu) and does not require any knowledge of the organism making the choice (internal milieu). The typical experimental paradigms used in cognitive neuropsychology are deterministic and veridical. (p. 365)

With some disappointment they concluded: “Paradoxically and almost incomprehensibly, the arsenal of cognitive neuroscience is virtually completely bereft of paradigms capable of examining how adaptive (as opposed to veridical) decisions are made” (p. 366). As a result, they called for innovative experimental procedures to determine the contribution of the prefrontal lobes to adaptive decision making.

Goldberg and Podell (1996) hit on a distinction closely related to one made in economics and decision theory: the distinction between risk and uncertainty (Knight, 1921). Risk, according to Knight, refers to situation of perfect knowledge: the decision maker knows the probabilities of all outcomes for all alternatives. This makes it possible to calculate the only correct, or optimal, response. Uncertainty, in contrast, refers to situations where the probabilities cannot be expressed with any mathematical precision, neither in frequencies nor in propensities. That is, in an uncertain world, the probabilities are unknown or unknowable. As an economist, Knight perceived this distinction to be important, since uncertainty may afford opportunities for profit that do not exist in situations where risks can be calculated (Rakow, 2010).

A related distinction was made by Savage (1954), known as the founder of modern Bayesian decision theory. Savage introduced the term “small worlds” for situations of perfect knowledge where all relevant alternatives, their consequences, and their probabilities are known for certain. According to him, these are the worlds in which Bayesian theory provides the best answer. Examples are lotteries and roulette. Small worlds need to be distinguished from “large worlds,” where part of the relevant information is unknown or must be estimated from small samples, or the future is uncertain (Savage, 1954; Binmore, 2009). Examples are decisions about when to plan a picnic, whom to marry, and how to raise your kids. Decision making under uncertainty is what our brain does most of the time, while situations of known risk are relatively rare and found mostly in gambling. Savage made it very clear that applying Bayesian theory to decisions in large (uncertain) worlds would be “utterly ridiculous” (p. 16) because there is no way to know all alternatives, consequences, and probabilities. As a consequence, the brain needs strategies beyond Bayes’ rule to succeed in an uncertain social and physical environment.

The distinction between risk and uncertainty has not always been recognized in cognitive neuroscience. In this article, we make a normative and a descriptive argument regarding this distinction:

1. *The best solution in a world of risk is generally not the best one in a world of uncertainty*. We argue that what the brain should do under risk does not necessarily generalize to what it should do under uncertainty.
2. *Cognitive processes in decisions under risk are not the same as in decisions under uncertainty*. We argue that cognitive processes observed under risk do not necessarily generalize to those the brain uses under uncertainty. Specifically, we argue:

*Risk:* Value-based statistical thinking (e.g., Bayesian probability updating plus utilities) is sufficient for making good decisions, provided that the problem is computationally tractable.

*Uncertainty:* Statistical thinking is no longer sufficient; heuristic thinking is required.

Much of cognitive neuroscience does not distinguish between risk and uncertainty. For instance, consider the claim, made in various forms, that the brain is Bayesian (e.g., Friston, 2010). Such a brain will likely provide optimal decisions only in small worlds, which are rare. Or consider the claim that there are two systems of reasoning: System 1, which is fast, heuristic, and prone to error, and System 2, which is slow, in keeping with the laws of probability, and rational (Sloman, 1996; Kahneman, 2011; for a critique, see Gigerenzer and Regier, 1996; Keren and Schul, 2009; Kruglanski and Gigerenzer, 2011). This two-system view does not consider that the laws of probability are sufficient for rationality in a small world only. In uncertain worlds, however, heuristics are indispensable. That is, both logic and heuristics are tools for different classes of problems. For instance, the recent financial crises illustrate that statistical tools for estimating risk, Bayesian or otherwise, failed consistently in the real, uncertain world of finance (Taleb, 2010). They are optimal when risks are known, but not in a world of uncertainty. Applying normative theories of risk to uncertain worlds can in fact lead to disasters. With respect to the financial crash of 2008, Stiglitz (2010) noted: “It simply wasn’t true that a world with almost perfect information was very similar to one in which there was perfect information” (p. 243). In sum, norms derived from assuming known risks do not simply generalize to norms under uncertainty.

## Rationality of Risk ≠ Rationality of Uncertainty

The point that the calculus of probability can determine the best action under risk but not under uncertainty is not new; it has been made as often by statisticians as it has been forgotten by cognitive scientists. Savage (1954) devoted the first half of his seminal book *Foundations of Statistics* to Bayesian decision theory, and the second half to heuristic decisions, such as minimax (choose the option that minimizes the maximal loss). Arrow (2004) similarly writes that in uncertain, ill-specified worlds, unbounded rationality (i.e., expected utility optimization) “has no meaning at all” (p. 54). What is new are scientific demonstrations that show that applying an optimization model to an uncertain world can lead to decisions that are normatively inferior to simple heuristics (see Gigerenzer et al., 2011). Here is an illustration:

### Mean-variance optimization leads to inferior results in the real world

#### Consider financial investment

A normative theory of how to allocate money to *N* assets is Markowitz’s Nobel prize-winning mean-variance model. Like all optimizing theories, it assumes a small world with perfect knowledge about the relevant parameters. Is this theory also optimal in the real, uncertain world of financial investment, where parameter values are not known for certain but need to be estimated? De Miguel et al. (2009) compared the mean-variance model with a heuristic called 1/*N*, or *equality heuristic*. The heuristic simply allocates money to *N* assets equally. The result was that 1/*N* consistently performed better in out-of-sample prediction (an elementary form of uncertainty). Cross-validation is a prime example of out-of-sample prediction: the data is divided into two complementary subsets: the in-sample data set, which is used for fitting the parameters of the competing models and an out-of-sample data set, which is used for testing how well the models predict (see also below). Note that in data fitting, that is, when all data are known, the optimizing model always wins, but not in prediction. None of 12 other optimization models, Bayesian or otherwise, could consistently predict better than the simple heuristic.

This result contradicts the widespread view that heuristics are always second best to logic and statistical optimization models. This view makes no distinction between risk and uncertainty. Researchers in this tradition have evaluated people’s reliance on 1/*N* negatively and attributed it to their cognitive limitations. However, ignoring part of the information is what makes heuristics robust for the unknown future, whereas by trying to integrate all information and estimate the weights, complex strategies such as the mean-variance portfolio suffer from overfitting the past. The mathematically sophisticated reader who wants to understand why and when simple heuristics can be more accurate than complex statistical methods will find an answer in the bias-variance dilemma (Gigerenzer and Brighton, 2009).

### The ecological rationality of simple heuristics

The fact that simple heuristics often outperform “optimization” models in situations of uncertainty has been demonstrated many times over (see Czerlinski et al., 1999; Gigerenzer and Brighton, 2009; Gigerenzer et al., 2011). In order to deal with an uncertain world, the brain relies on an *adaptive toolbox* of heuristics. Accordingly, intelligence is defined as the degree of knowing in which situation to use which heuristic. The scientific study of this normative question is called the study of the *ecological rationality* of a heuristic. For instance, 1/*N* tends to outperform mean-variance optimization in situations where predictive uncertainty is high (stocks are hard to predict), the number of options *N* is large (the optimization models have to estimate more parameters which leads to more error), and the sample size is relatively small. In uncertain worlds with these features, 1/*N* can be expected to be both faster and more accurate than the mean-variance optimization. When would mean-variance outperform 1/*N*? De Miguel et al. (2009) estimated that with 50 assets, one would need some 500 years of stock data before the optimization model is profitable.

Humans rely on the 1/*N* heuristic not only for financial investment. Many parents who have two or more children try to distribute their time and love equally. For three or more children, this heuristic paradoxically predicts interesting inequalities in the long run because the first and last-born get more time, dependent on the spacing between births. Tests have provided empirical evidence for these predictions (Hertwig et al., 2002). In many situations, fairness und justice are achieved by distributing resources equally.

Our normative argument has fundamental consequences for the neuroscience of decision making: Claims that the rational brain always works by Bayesian calculations are founded on the assumption that what is rational in a world of risk is also rational in an uncertain world – the world our brain has to deal with most of the time. These claims are also incompatible with three well-known restrictions: Bayesian optimization is not feasible if (i) the choice alternatives are not known for sure, (ii) the mind has more than one goal, and (iii) even if all alternatives were known and the mind had only one goal, the calculations can quickly become computationally intractable, that is, no mind can actually perform them in a lifetime (Gigerenzer, 2004). Bayesian inference works in small worlds where there are reliable data for probabilities and only a few alternatives and cues.

## Cognitive Processes in Situations of Risk ≠ Processes in Situations of Uncertainty

In the previous section, we argued that what is optimal in a world of risk is typically not the best in a world of uncertainty. Consequently, an adapted brain relies on different processes according to the situation. When faced with risk, using heuristics is of little value, unless the computations become too difficult. When faced with uncertainty, using logic and statistics is of little value, unless the part of the problem that is known is being calculated.

We would like to emphasize the importance of the distinction between risky and uncertain worlds for the neuroscientific investigation of decision making. So far, its focus has been on small world problems. But just as normative results from studying cognition in small worlds do not automatically generalize to what people should do in uncertain worlds, we cannot be sure that descriptive results generalize either. Influenced by small-word theories of decision making, neuroscientists, and neuroeconomists have nevertheless relied heavily on the “gambling paradigm” as a model for exploring the neural correlates. In a typical neuroeconomic paradigm, participants are presented with the choice between two options, Option A and Option B, which differ with respect to objective dimensions such as the magnitude and the probability of reward (as assigned by the experimenter). Reward is largely defined as monetary value, which the participant will receive after the functional session. These problems require entirely different skills and strategies than decisions under uncertainty. For example, although calculating the expected value might suffice for a lottery, it will not be sufficient for deciding whether to be vaccinated against swine flu, which share to buy, or whom to marry.

Results such as the finding that “activity in the ventral striatum during the evaluation of monetary gambles is non-linear in probabilities in the pattern predicted by prospect theory” (Hsu et al., 2009, p. 2231) may capture the neural activation pattern when comparing gambles. Concluding that this activity pattern will also be observed when searching for jobs or mates, however, is not warranted. The pattern predicted by prospect theory in fact disappears and even reverses when the probabilities are not provided by the experimenter but the participant instead has to learn these from experience, a phenomenon known as the description-experience gap (Hertwig and Erev, 2009). Nor do findings from small worlds easily translate into a cognitive process model, that is, testing for the neural correlates of some form of utility model cannot elucidate the cognitive mechanisms in large world problems. In the words of Colombo and Seriès (2012): “that the brain *is* a Bayesian machine does not follow from the fact that Bayesian models are *used* to study the brain and the behavior it generates” (p. 2).

In an uncertain world, there is broad experimental evidence that humans and other animals rely on a toolbox of heuristics. These are based on evolved and learned core capacities and include (for details, see Gigerenzer and Gaissmaier, 2011):

– *Recognition-based heuristics:* Recognition heuristic (RH; see below); fluency heuristic^1^.
– *Equality-based heuristics*: 1/*N* (see above); tallying (weight reasons equally).
– *One-good-reason heuristics:* take-the-best (see below); fast-and-frugal trees^2^.
– *Social heuristics*: tit-for-tat; imitate-the-majority.

What would a neuroscientific investigation of heuristic decision making look like? One approach is to study the neural correlates of heuristic processes, such as search rules, stopping rules, and decision rules (e.g., Volz et al., 2006, 2010; Khader et al., 2011; Rosburg et al., 2011). In what follows, we provide two illustrations for how to go beyond lotteries and study the neural correlates of the use of cognitive heuristics in an uncertain world.

## Neural Correlates of Heuristic Decisions in Uncertain Worlds

Note that studying decision making under uncertainty (as opposed to risk) does not require squeezing the complexity of the large world into the laboratory. It simply requires studying tasks where not all alternatives, consequences, and probabilities are known for sure or provided by the experimenter.

### Recognition heuristic

Consider a simple heuristic that humans and other animals use to make inferences about an uncertain world (Goldstein and Gigerenzer, 2002):

*Recognition heuristic:* If one of two objects is recognized and the other is not, then infer that the recognized object has the higher value with respect to the criterion.

For instance, consider the question whether Milan or Modena has more inhabitants. If one has heard of Milan but not of Modena, the inference is that Milan is the larger city. Note that the RH requires semi-ignorance to be *applicable*, meaning that if one has heard of both (or neither) objects, it will not be effective. Experimental studies indicate that a large proportion of subjects rely on it in uncertain situations, such as when predicting which tennis player will win in Wimbledon or which political candidate to vote for, and by animals when choosing food (Gigerenzer and Goldstein, 2011). These studies report a substantial correlation between the proportion of judgments that follow the RH and the validity of recognition for the task, suggesting an adaptive use of the heuristic.

There are two competing hypotheses in the literature: that people use the RH in an adaptive or in an automatic way. The adaptive use requires two processes. The first assesses whether or not the alternatives are recognized and hence whether the RH *can* be applied in principle. The second process assesses whether the RH *should* be applied, which is essentially a judgment about the heuristic’s ecological rationality, that is, the match between mind and environment. In contrast, the automatic use entails only one process: automatically choosing the recognized alternative, without considering why recognition should be predictive of the criterion. Such an automatic strategy would also be successful for the Milan–Modena question, where recognition is so highly correlated with city size.

In 2006, we tested these hypotheses with the help of functional magnetic resonance imaging (fMRI; Volz et al., 2006). To see whether RH-based decision processes depend on additional judgments of ecological rationality, which should draw on brain areas beyond those known to reflect recognition memory processes, we ran two experiments. In experiment 1, participants were presented with the names of two cities and asked to indicate which city in each pair is larger (recognition plus inference). In experiment 2, participants were presented with the names of two cities and asked to indicate which city they knew in each pair (recognition only). Comparing the activation results of the two experiments, we found that decision processes in both experiments 1 and 2 drew on medial parietal areas, which are assumed to reflect recognition memory processes. In contrast, specifically RH-based decision processes (in experiment 1) drew additionally on the anterior medial prefrontal cortex, which is taken to reflect judgments of the ecological rationality of the RH in terms of assessing one’s own sense of recognition. Thus, RH-based decision processes go beyond automatically choosing the recognized alternative and are guided by judgments about the ecological rationality of the RH, as reflected by activation in anterior medial prefrontal cortex.

The study illustrates how fMRI can be used to compare competing hypotheses about the selection of heuristics: here, hypotheses on automatic versus adaptive use.

### Take-the-best heuristic

The RH draws on the core capacity of recognition of names, faces, or other stimuli. If both objects are recognized, the RH is not applicable, but the take-the-best heuristic (TTB) is. Like the RH, take-the-best models how people infer which of two objects has a higher value on a criterion based on cue values retrieved from memory (Gigerenzer and Goldstein, 1996). The heuristic is defined by three building blocks:

Take-the-best heuristic:

(i) *Search rule*: search through cues according to their validity.
(ii) *Stopping rule*: stop search on finding the first cue that discriminates between the objects.
(iii) *Decision rule*: infer that the object with the positive cue value has the higher criterion value.

Thus, according to this cognitive process model, information search is terminated as soon as a cue discriminates between the alternatives; other cues are not activated. For instance, if a person has heard of both Milan and Modena and recalls that Milan is a state capital (the most valid cue) but Modena is not, that person would stop search for further cues and infer that Milan has the larger population.

Note that take-the-best implies a lexicographic step-by-step process with limited search. This process is quite different from weighting-and-adding all cues, which is assumed in models that postulate the integration of all cues, such as in value-based decision models. Experimental studies have provided strong evidence that many people’s memory-based inferences are consistent with the predictions of take-the-best (and inconsistent with those of adding-and-weighting models) in situations where its use is ecologically rational (e.g., Rieskamp and Otto, 2006; Bröder, 2011). Specifically, experts appear to rely on simple search and stopping rules more often than novices (Garcia-Retamero and Dhami, 2009).

Can cognitive neuroscience provide evidence for the hypothesis of limited search, as defined in the stopping rule of take-the-best? Khader et al. (2011) used fMRI to test the assumption that heuristics simplify decision making by activating long-term memory representations of only those attributes that are necessary for the decision, since it is unclear from behavioral studies alone whether using heuristics is indeed associated with limited memory search (with the exception of reaction time studies; see Bröder and Gaissmaier, 2007). Accordingly, the authors monitored the activation of specific long-term memory representations while participants made memory-based decisions using the take-the-best heuristic.

Khader et al. (2011) taught their subjects to make decisions using the TTB heuristic while measuring their hemodynamic response. Particularly, they let their participants first learn by trial and error to associate each of 16 fictional company names with a specific stimulus pattern of four binary cues (objects, houses, locations, faces). Then, participants learned how to make decisions using the TTB heuristic for a fictional job selection scenario (i.e., which of two applicants is more suitable for a job). Thereafter, participants learned by trial and error (i) the importance of the different attributes for predicting which of two companies would be more successful, e.g., the attribute hierarchy objects > houses > locations > faces; and (ii) which stimulus was predictive of higher success, that is, the attribute direction. In each phase, participants learned until they satisfied a criterion.

In the actual decision making task, participants were presented with only the names of two companies and then had to infer, by using the TTB heuristic, which company will be more successful in the next year. To do so, participants had to retrieve all the relevant attribute information from long-term memory. The attributes with which the two companies were described consisted of visual information known to be represented in different parts of the posterior cortex, e.g., in a face-specific and in a house-specific region of interest. That allowed the authors to examine activation within these regions of interest as a function of the number of the to-be-retrieved attributes. Given the cognitive process model of the TTB heuristic, Khader et al. (2011) expected the activation in the regions of interest to be systematically modulated by the relative importance of the information for making a decision. Their specific analyses revealed a controlled retrieval shown by a selective boosting of activation, specifically in those regions that represent the attributes that were relevant for the decision. For example, activation strongly increased in the face-specific region solely in those trials in which faces were relevant for the decision. Furthermore, a prolonged response to an attribute was found only when it was relevant late in the decision process, when the attribute was low in importance. All in all, the data showed a “selective modulation of neural activation that follows the retrieval order according to TTB” (p. 11), which the authors take to support the notion of controlled retrieval processes.

Thus, by using fMRI the authors could provide evidence in favor of the cognitive process model’s prediction for the decision phase, i.e., using the TTB heuristic is indeed associated with a controlled activation of decision-relevant attribute representations. As in the case of the RH, the imaging study was used to compare two competing models, exhaustive search as assumed in standard weighting-and-adding models and limited search as defined by take-the-best.

## Study the Neural Correlates of Process Models, Not as-if Models

Why are experimental studies with situations of known risk, such as lotteries, so popular? This is especially puzzling given that lotteries, roulette, and other tasks with known risks are quite a recent in human history. One answer is that they facilitate application of statistical optimization models, such as expected utility and Bayesian updating, which are also quite recent achievements in human history. However, studies of cognitive processes have provided little evidence that the mind engages in expected utility calculations during decision making; instead, there is reliable evidence for the use of heuristics (see Ford et al., 1989; Payne et al., 1993; Friedman and Sunder, 2011). For instance, Friedman and Sunder (p. 1) concluded in their review of the literature on risky choice from 1950 to 2010:

No such functions [utility or similar Bernoulli functions] have yet been found that are useful for out-of-sample predictions. Nor do we find practical applications of Bernoulli functions in major risk-based industries such as finance, insurance, and gambling.

The important methodological concept is “out-of-sample prediction.” Expected utility theory or its variants such as prospect theory can easily fit their parameters to the data after the fact, but the real test is in prediction, not fitting. “Out-of-sample” means that the parameters of a model, heuristic, or optimizing, are fitted to one part (e.g., half) of the sample and the other part is tested. This is an elementary form of uncertainty, where not all data are known. Most importantly, neoclassical economists have never claimed that the brain computes expected utilities but explicitly emphasize that optimization models do not describe the cognitive process. Following D:Friedmans:1953] as-if methodology, economists consider these models only as tools for prediction, making deliberately “wrong” assumptions that are mathematically convenient. Unfortunately many cognitive neuroscience studies appear to be unaware of this conceptual problem and search for the neural correlates of “as-if” models.

## Conclusion

We distinguished between two kinds of problems humans face: worlds of *risk* or worlds of *uncertainty*. In a world of risk (small world), all relevant alternatives, their probabilities, and their consequences are known for sure and the future is certain. In contrast, in a world of uncertainty (large world) part of the information is unknown or has to be estimated from small samples, and surprises can happen. The second distinction we introduced is between *what* decisions people make (the outcome) and *how* they make them (the process). Answering the first question leads to *as-if* models; answering both questions leads to *process* models. We argue that the two distinctions are correlated: As-if models tend to match small world studies, whereas process models tend to match large world studies.

We pointed out the strong focus on decision making under risk in neuroscientific studies, which pay little attention to how the brain makes adaptive decisions in an uncertain world. That becomes problematic when the normative and descriptive results are generalized to how the brain deals with an uncertain world. In addition, we provided evidence that the normative solution under risk is not the best one under uncertainty. We also provided evidence that the cognitive processes for decisions in a world of risk are not the same as in a world of uncertainty. The study of behavior in lotteries – and other small world tasks – does not address the question of how humans make decisions when the conditions for rationality postulated by the model of neoclassical economics are not met, a question emphasized by Simon (1989). In large worlds, people cannot optimize but instead “satisfice” by relying on the brain’s adaptive toolbox.

In sum, the current focus of cognitive neuroscience studies on situations where all risks are known and optimization is possible imposes limits on the understanding of adaptive brain processes, both normatively and descriptively. The neural correlations of cognitive processes such as heuristic search, stopping rules, and aspiration levels have little chance of being detected and may even be taken for correlates of expected utility and other as-if theories.

## Conflict of Interest Statement

The authors declare that the research was conducted in the absence of any commercial or financial relationships that could be construed as a potential conflict of interest.

## Footnotes

^1^The fluency heuristic is a simple heuristic that can be used to exploit recognition memory and is defined in the following way: If two objects are recognized, and one of objects is more fluently retrieved, then infer that this object has the higher value with respect to criterion; where retrieval fluency is defined as how long it takes to retrieve a trace from long-term memory (c.p. Schooler and Hertwig, 2005).

^2^Fast-and-frugal decision trees are simple rules for categorization; they are fast-and-frugal since they allow a classification decision at each level of the tree (c.p. Martignon et al., 2011). For binary predictors, a fast-and-frugal tree has *n* + 1 exists, while a full tree has 2*^n^* exits. An example is the Simple Triage and Rapid Treatment (START) procedure, which is used to categorize patients into those who need immediate medical treatment and those whose treatment can be delayed (Super, 1984). By using the START, “a paramedic sequentially checks up to five diagnostic cues to decide which category a person falls into; a decision can be made after each cue is checked” (Luan et al., 2011, p. 316). By using such a simple and transparent decision tree, the decision maker/paramedic does not need to search for and integrate all the relevant cues so as to reach a sound decision.
